# On an Implicit Model Linear in Both Stress and Strain to Describe the Response of Porous Solids

**DOI:** 10.1007/s10659-021-09831-x

**Published:** 2021-04-14

**Authors:** Hiromichi Itou, Victor A. Kovtunenko, Kumbakonam R. Rajagopal

**Affiliations:** 1 Department of Mathematics, Tokyo University of Science, 1-3 Kagurazaka, Shinjuku-ku Tokyo, 162-8601 Japan; 2 Institute for Mathematics and Scientific Computing, University of Graz, NAWI Graz, Heinrichstr.36, 8010 Graz, Austria; 3 Lavrentyev Institute of Hydrodynamics, Siberian Division of the Russian Academy of Sciences, 630090 Novosibirsk, Russia; 4 Department of Mechanical Engineering, Texas A&M University, College Station, TX 77843 USA

**Keywords:** Nonlinear elasticity, Porous metals, Implicit material response, Volumetric-deviatoric decomposition, Mixed variational formulation, Quasi-linear elliptic problem, Linear-fractional singularity, Regularization, Thresholding, Well-posedness, 74B20, 35Q74, 47J07, 49J52

## Abstract

We study some mathematical properties of a novel implicit constitutive relation wherein the stress and the linearized strain appear linearly that has been recently put into place to describe elastic response of porous metals as well as materials such as rocks and concrete. In the corresponding mixed variational formulation the displacement, the deviatoric and spherical stress are three independent fields. To treat well-posedness of the quasi-linear elliptic problem, we rely on the one-parameter dependence, regularization of the linear-fractional singularity by thresholding, and applying the Browder–Minty existence theorem for the regularized problem. An analytical solution to the nonlinear problem under constant compression/extension is presented.

## Introduction

When the implicit relation between the stress $\mathbf{T}$, deformation gradient $\mathbf{F}$ and density $\rho $ introduced by [20, 21] to describe the response of elastic bodies, is linearized by assuming that the displacement gradient is small, it reduces to

1 $$ \beta _{0}\boldsymbol{\varepsilon } +\beta _{1}\mathbf{I} +\beta _{2} \mathbf{T} +\beta _{3}\mathbf{T}^{2} +\beta _{4}(\mathbf{T} \boldsymbol{\varepsilon } +\boldsymbol{\varepsilon }\mathbf{T}) +\beta _{5}( \mathbf{T}^{2}\boldsymbol{\varepsilon } +\boldsymbol{\varepsilon } \mathbf{T}^{2}) =\mathbf{0}, $$

where $\mathbf{I}$ is the identity transformation, $\boldsymbol{\varepsilon }$ is the linearized strain, the $\beta _{i}$, $i=1,2,3$ are scalar valued functions that can at most depend linearly on $\boldsymbol{\varepsilon }$, but arbitrarily on the invariants of $\mathbf{T}$, while $\beta _{i}$, $i=0,4,5$ depends on the invariants of $\mathbf{T}$. Since by virtue of the balance of mass: $\rho _{R} =\rho ({\mathrm{det}}\mathbf{F})$, which when linearized leads to $\rho _{R} =\rho (1 +{\mathrm{tr}}\boldsymbol{\varepsilon })$, ${\mathrm{tr}}\boldsymbol{\varepsilon }$ can be replaced by $\rho $, which makes the constitutive relation useful in describing the response of porous materials as the porosity determines the density of the material (see [23] for discussion of the development and relevance of such constitutive relations). Gainfully exploiting the fact that such constitutive relations can accommodate the material moduli can be functions of the density, [18, 19] studied the problem of initiation of damage in concrete.

A subclass wherein the constitutive relation is also linear in $\mathbf{T}$ is given by

2 $$ (1 +\lambda _{3} {\mathrm{tr}}\mathbf{T}) \boldsymbol{\varepsilon } =E_{1} (1 +\lambda _{1} {\mathrm{tr}}\boldsymbol{\varepsilon }) \mathbf{T} +E_{2} (1 + \lambda _{2} {\mathrm{tr}}\boldsymbol{\varepsilon }) ({\mathrm{tr}}\mathbf{T}) \mathbf{I}. $$

In (2) moduli $\lambda _{1}$, $\lambda _{2}$, $\lambda _{3}$, $E_{1}$, $E_{2}$ are all constants. When $\lambda _{1}$, $\lambda _{2}$, $\lambda _{3}$ are all zero, we recover the equation for classical linearized elasticity. Then, we can identify

$$ E_{1} =\frac{1+\nu }{E} =\frac{1}{2\mu } >0,\quad E_{2} =-\frac{\nu }{E} < 0, $$

where $E$ is the Young’s modulus and $\nu $ is the Poisson’s ratio, which are related to the Lame’ constants $\lambda $ and $\mu $. Let us express the stress in terms of its deviatoric and spherical parts by using

$$ \mathbf{T} =\mathbf{T}^{*} +\frac{1}{3} ({\mathrm{tr}}\mathbf{T}) \mathbf{I}. $$

Then (2) reduces to the equation

3 $$ (1 +\lambda _{3} {\mathrm{tr}}\mathbf{T}) \boldsymbol{\varepsilon } =E_{1} (1 +\lambda _{1} {\mathrm{tr}}\boldsymbol{\varepsilon }) \mathbf{T}^{*} +E_{4} (1 +\lambda _{4} {\mathrm{tr}}\boldsymbol{\varepsilon }) ({\mathrm{tr}}\mathbf{T}) \mathbf{I}, $$

where the new coefficients $E_{4}$ and $\lambda _{4}$ are expressed as

$$ E_{4} =\frac{E_{1}}{3} +E_{2} =\frac{1-2\nu }{3E} =\frac{1}{9K} >0, \quad \lambda _{4} = \frac{(E_{1}/3) \lambda _{1} + E_{2} \lambda _{2}}{E_{4}} $$

using the bulk modulus $K$.

Earlier studies that are relevant to the analysis considered here are the investigations into bodies exhibiting limiting small strain in a nonlinear elastic body by [7], on viscoelastic bodies by [8, 11, 12], and with regard to implicitly constituted quasi-linear viscoelastic bodies by [9]. A relevant subclass of contact problems in the bodies with non-penetrating cracks was developed by [14].

The general implicit model relating the Cauchy–Green tensor and the Cauchy stress has no issues concerning frame-indifference. Of course, when we linearize since the linearized strain is not frame indifferent, the linearization of the implicit model will not be frame-indifferent.

While it is true that our model shares negative aspects with the classical linearized model with regard to singularities of the linearized strain, the general implicit constitutive relations when linearized also leads to strain-limiting models for the linearized strain (the strain can be fixed apriori to be as small as one wants) and in the case of such models very rigorous mathematical results have been established. Existence of weak solutions to fully three-dimensional problems for a class of limiting strain models is presented in [3], and the existence of solutions to anti-plane problem for weak solutions of a class of strain limiting models can be found in [4]. Our model shares other negative aspects with the classical linearized model giving rise to singularities at corners, etc., however it has certain useful features that the classical linearized elastic model does not have as documented in the next comment.

In our model, the term $E_{1} (1 +\lambda _{1} {\mathrm{tr}}\boldsymbol{\varepsilon })$ can be viewed as a density dependent material moduli as ${\mathrm{tr}}\boldsymbol{\varepsilon }$ can be expressed in terms of the density. Thus, our model is a linearized model that can describe the small displacement gradient response of porous materials whose material moduli would depend on the density. In a porous material we expect the material moduli to be density dependent. The material moduli of a classical linearized elastic model are constants.

As we mentioned earlier, by virtue of the balance of mass, we can in fact rewrite the constitutive relation (2) by introducing the density in the place of the ${\mathrm{tr}}\boldsymbol{\varepsilon }$. On taking the trace of (3) we get the implicit function

4 $$ \boldsymbol{\Phi }({\mathrm{tr}}\boldsymbol{\varepsilon }, {\mathrm{tr}} \mathbf{T}) := {\mathrm{tr}}\boldsymbol{\varepsilon } -3E_{4} {\mathrm{tr}} \mathbf{T} +(\lambda _{3} -3E_{4} \lambda _{4}) ({\mathrm{tr}} \boldsymbol{\varepsilon }) {\mathrm{tr}}\mathbf{T} =0, $$

because ${\mathrm{tr}}\mathbf{T}^{*} =0$, and ${\mathrm{tr}}\mathbf{I} =3$ in 3d. The equation (4) can be solved explicitly with respect to the trace of the linearized strain as

5 $$ {\mathrm{tr}}\boldsymbol{\varepsilon } = \frac{3E_{4} {\mathrm{tr}}\mathbf{T}}{1+(\lambda _{3} -3E_{4} \lambda _{4}) {\mathrm{tr}}\mathbf{T}}. $$

It is important to bear in mind that the ${\mathrm{tr}}\boldsymbol{\varepsilon }$ has to be small and hence the right hand side of (5) has to be small. That is $E_{4}$, the $\lambda $s, and the ${\mathrm{tr}}\mathbf{T}$ have to be such that this is true.

Using equation (4) one can also obtain an expression for the Cauchy stress in term of the linearized strain. This would however involve terms that are higher order in the linearized strain which we have already neglected. One has to be careful in dealing with approximations that stem from the implicit constitutive relations that lead to equation (1). It might be possible to express the stress as a nonlinear function of the linearized strain, but this cannot be viewed as a constitutive relation. One ought to always consider the problem wherein the linearized strain appears only linearly in the approximate constitutive relation.

The following remarks make this clear. Suppose one considers a special sub-class of the implicit expression that expresses the Cauchy–Green tensor $\mathbf{B}$ as a function of the Cauchy stress. When one linearizes the same assuming that the displacement gradient is small, then one obtains an approximation wherein the linearized strain is a nonlinear function of the stress. Inverting it could, and most often would, lead to the stress as a nonlinear function of the linearized strain which is not allowable according to our basis for the approximation in the first place. The point is, when dealing with implicit constitutive relations if it is possible to express the Cauchy–Green tensor in terms of the stress, then inverting this expression if this is possible and linearizing is not the same as linearizing and inverting (see [22] for a detailed discussion of the same). We should always use the expression wherein the linearized strain occurs linearly as the appropriate form of the approximate constitutive relation.

Inserting (5) into (2) and dividing the result by $1 +\lambda _{3} {\mathrm{tr}}\mathbf{T}$ it follows that

6 $$\begin{array}{c} \varepsilon =E_{1}\frac{1+\left(\lambda_{3}+3E_{4}(\lambda_{1}-\lambda_{4})\right)\mathrm{tr}T}{(1+\lambda_{3}\mathrm{tr}T)\left(1+(\lambda_{3}-3E_{4}\lambda_{4})\mathrm{tr}T\right)}T\\ +E_{2}\frac{1+\left(\lambda_{3}+3E_{4}(\lambda_{2}-\lambda_{4})\right)\mathrm{tr}T}{(1+\lambda_{3}\mathrm{tr}T)\left(1+(\lambda_{3}-3E_{4}\lambda_{4})\mathrm{tr}T\right)}(\mathrm{tr}T)I. \end{array}$$

**Fig. 1:**
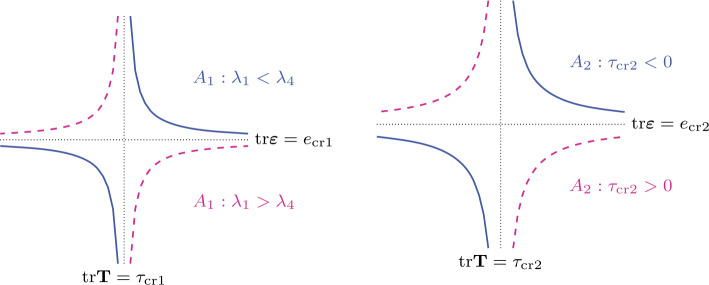
Linear-fractional functions $A_{1}$ (left plot) and $A_{2}$ (right plot)

Splitting the stress into its deviatoric and spherical parts according to (3), and taking into consideration that

$$ E_{1} \frac{1 +\bigl( \lambda _{3} +3 E_{4} (\lambda _{1} -\lambda _{4}) \bigr) {\mathrm{tr}}\mathbf{T}}{3} +E_{2} \Bigl( 1 +\bigl( \lambda _{3} +3 E_{4} (\lambda _{2} -\lambda _{4}) \bigr) {\mathrm{tr}}\mathbf{T} \Bigr) =E_{4} (1 +\lambda _{3} {\mathrm{tr}} \mathbf{T}), $$

we express the response function (6) equivalently in the form:

7 $$ \boldsymbol{\varepsilon } =E_{1} A_{1}({\mathrm{tr}}\mathbf{T}) A_{2}({\mathrm{tr}} \mathbf{T}) \mathbf{T}^{*} +E_{4} A_{2}({\mathrm{tr}}\mathbf{T}) ({\mathrm{tr}} \mathbf{T}) \mathbf{I}, $$

where the linear-fractional factors $A_{1}$ and $A_{2}$ are defined by

$$ A_{1}({\mathrm{tr}}\mathbf{T}) :=1 + \frac{3E_{4} (\lambda _{1} -\lambda _{4})}{1/{\mathrm{tr}}\mathbf{T} -1/\tau _{\mathrm{cr1}}},\quad A_{2}({\mathrm{tr}} \mathbf{T}) :=\frac{1}{1 -{\mathrm{tr}}\mathbf{T}/\tau _{\mathrm{cr2}}}, $$

with $\tau _{\mathrm{cr1}} :=-1/\lambda _{3}$ and $\tau _{\mathrm{cr2}} :=1/(3 E_{4} \lambda _{4} -\lambda _{3})$. For example, we portray in the left and right plots of Fig. 1, respectively, $A_{1}$ and $A_{2}$ from (7) in the $({\mathrm{tr}}\mathbf{T}, {\mathrm{tr}}\boldsymbol{\varepsilon })$-coordinates with regard to their dependence of the sign of $\lambda _{1} -\lambda _{4}$ and $\tau _{\mathrm{cr2}}$. It is worth noting that the sign and order relations between $\tau _{\mathrm{cr1}}$ and $\tau _{\mathrm{cr2}}$, between $e_{\mathrm{cr1}} :=-1/\lambda _{4}$ and $e_{\mathrm{cr2}} :=(3E_{4})/(\lambda _{3} -3E_{4} \lambda _{4})$ are arbitrary.

We conclude that linear-fractional functions $A_{1}$ and $A_{2}$ are neither bounded from below nor above, and not even continuous so that no well-posedness theory can be applied to the constitutive relation (7). Therefore, assuming $\lambda _{1} =\lambda _{3} =0$ such that the factor $A_{1} A_{2} =1$ in front of $\mathbf{T}^{*}$, we regularize by thresholding the unbounded and discontinuous function $A_{2}({\mathrm{tr}}\mathbf{T}) {\mathrm{tr}}\mathbf{T} =:B({\mathrm{tr}}\mathbf{T})$. Then we apply the existence theorem for monotonous, bounded, coercive, and hemi-continuous operators (see [2, 17]). If $\lambda _{1}$ is set to be zero, then the material moduli cannot depend on the mean value of the stress, that is the mechanical pressure. Even in the case of pressure dependent viscosity of the Navier–Stokes fluid, unless the viscosity is also dependent on the shear rate, it is not possible to establish existence results (see [6, 15, 16]).

## One-Parameter Regularized Model

When we set $\lambda _{1} =\lambda _{3} =0$ the constitutive relation (6) reduces to

$$ \boldsymbol{\varepsilon } =E_{1} \mathbf{T} +E_{2} \frac{1 +3 E_{4} (\lambda _{2} -\lambda _{4}){\mathrm{tr}}\mathbf{T}}{ 1 -3E_{4} \lambda _{4} {\mathrm{tr}}\mathbf{T}} ({\mathrm{tr}}\mathbf{T}) \mathbf{I}, $$

and with the help of (7) it takes the equivalent form:

8 $$ \boldsymbol{\varepsilon } =E_{1} \mathbf{T}^{*} +E_{4} \frac{{\mathrm{tr}}\mathbf{T}}{1 -3E_{4} \lambda _{4} {\mathrm{tr}}\mathbf{T}} \mathbf{I}. $$

Let us decompose the stress into two independent variables as

9 $$ \mathbf{T} =\mathbf{T}^{*} +\frac{1}{3} p \mathbf{I},\quad {\mathrm{tr}} \mathbf{T}^{*} =0, $$

where $p :={\mathrm{tr}}\mathbf{T}$. With the help of (9) and using

10 $$ \boldsymbol{\varepsilon } =\boldsymbol{\varepsilon }^{*} +\frac{1}{3} ({ \mathrm{tr}}\boldsymbol{\varepsilon }) \mathbf{I}, $$

equation (8) is decoupled into the deviatoric and spherical parts as

11 $$ \boldsymbol{\varepsilon }^{*} =E_{1} \mathbf{T}^{*},\quad \frac{1}{3} { \mathrm{tr}}\boldsymbol{\varepsilon } =E_{4} B(p),\quad B(p) := \frac{p}{1 -p/\tau _{\mathrm{cr}}},\quad \tau _{\mathrm{cr}} := \frac{1}{3E_{4} \lambda _{4}}. $$

**Fig. 2:**
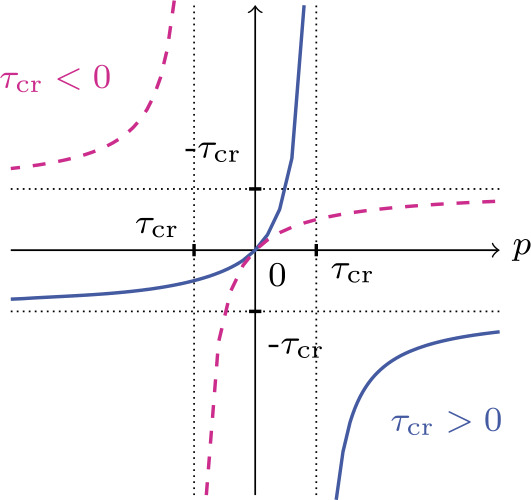
Linear-fractional function $B(p)$ depending on the sign of $\tau _{\mathrm{cr}}$

In Fig. 2 we portray the linear-fractional function $B$ from (11) versus $p$ with the regions wherein the signs $\tau _{\mathrm{cr}}$ are delineated. Note that $B(0) =0$.

**Fig. 3:**
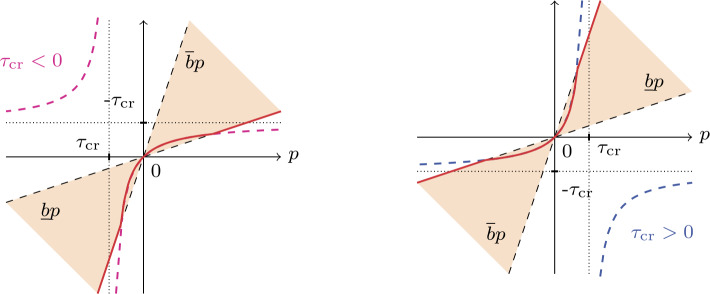
The threshold function $\mathcal{F}[B(p)]$ for $\tau _{\mathrm{cr}}<0$ (left plot) and $\tau _{\mathrm{cr}}>0$ (right plot)

For $\underline{b}$ and $\overline{b}$ chosen as positive thresholds such that $0< \underline{b}< \overline{b}< \infty $, we regularize the discontinuous function $B$ in (11) by piecewise functions

12 $$ \mathcal{F}[B(p)] := \textstyle\begin{cases} B(p) & \text{if } 1-1/\underline{b} < p/\tau _{\mathrm{cr}}< 1-1/ \overline{b}, \\ \overline{b} p & \text{if } p/\tau _{\mathrm{cr}}\ge 1-1/\overline{b}, \\ \underline{b} p & \text{if } p/\tau _{\mathrm{cr}}\le 1-1/\underline{b}, \end{cases} $$

which is comprised of three pieces. The example $\mathcal{F}[B(p)]$ from (12) is portrayed in the left and right plots of Fig. 3 depending on either $\tau _{\mathrm{cr}}<0$ or $\tau _{\mathrm{cr}}>0$. In this example, $0< \underline{b}<1$ guarantees that the point $p/\tau _{\mathrm{cr}} =1-1/\underline{b}<0$, and the choice $1< \overline{b}$ guarantees the other point $p/\tau _{\mathrm{cr}} =1-1/\overline{b}>0$.

### Lemma 1

(Threshold linear-fractional function)

*The regularized function* $p\mapsto \mathcal{F}[B(p)]$ *in* (12) *is continuous*, *coercive and bounded such that*

13 $$ \mathcal{F}[B(p)] p\ge \underline{b} p^{2},\quad \bigl| \mathcal{F}[B(p)] \bigr|\le \overline{b} |p|, $$

*and strongly monotone*:

14 $$ \bigl( \mathcal{F}[B(p)]- \mathcal{F}[B(q)] \bigr) (p-q) \ge \min ( \underline{b}, \underline{b}^{2}) (p-q)^{2}. $$

### Proof

From (11) we calculate that $B(p) =\underline{b} p$ at $p/\tau _{\mathrm{cr}}= 1-1/\underline{b}$, and similarly $B(p) =\overline{b} p$ at $p/\tau _{\mathrm{cr}}= 1-1/\overline{b}$. Therefore, the piecewise-continuous function from (12) at these two points is continuous.

From (12) we conclude that $\mathcal{F}[B(p)]$ lies in the cone:

15 $$ \min (\underline{b} p, \overline{b} p)\le \mathcal{F}[B(p)]\le \max ( \underline{b} p, \overline{b} p), $$

which leads to (13). Since $B^{\prime }(p) =1/(1 -p/\tau _{\mathrm{cr}})^{2} >0$ for $p\neq\tau _{\mathrm{cr}}$, each branch of $B$ as $p<\tau _{\mathrm{cr}}$ and $p>\tau _{\mathrm{cr}}$ is monotone, and composed continuously in (12) with the two linear pieces $\underline{b} p$ and $\overline{b} p$ it remains monotone. The derivative bounds $\min (\underline{b}, \underline{b}^{2})\le (\mathcal{F}[B(p)])^{\prime }\le \max (\underline{b}, \underline{b}^{2})$ follow the lower estimate (14). □

Using the result of Lemma 1, in the next section we formulate the nonlinear elasticity problem for three independent variables $\boldsymbol{\varepsilon }$, $\mathbf{T}^{*}$ and $p$. The mixed three-field formulation of linear elastic model was used for the reason of FEM analysis in [1, 5]. For a constitutive equation wherein the material moduli depend on the mean normal stress, see [10, 13].

## Analysis of the Governing Equations Using the Three Fields $\boldsymbol{\varepsilon }$, $\mathbf{T}^{*}$ and $p$ as the Variables

Let $\Omega $ be a bounded domain in the Euclidean space $\mathbb{R}^{3}$ with the Lipschitz continuous boundary $\partial \Omega $ and the unit normal vector $\mathbf{n} =(n_{1},n_{2},n_{3})$ which is directed outward. We assume that $\partial \Omega =\overline{\Gamma _{\mathrm{N}}}\cup \overline{\Gamma _{\mathrm{D}}}$ consists of two disjoint parts: the Neumann boundary $\Gamma _{\mathrm{N}}$ and the nonempty Dirichlet boundary $\Gamma _{\mathrm{D}}$.

For spatial points $\mathbf{x}=(x_{1},x_{2},x_{3})$ in $\overline{\Omega }$, let the body force $\mathbf{f} =(f_{1},f_{2},f_{3})(\mathbf{x})$ for $\mathbf{x}\in \Omega $ and the boundary traction $\mathbf{g} =(g_{1},g_{2},g_{3})(\mathbf{x})$ for $\mathbf{x}\in \Gamma _{\mathrm{N}}$ be given. We look for the displacement vector $\mathbf{u} =(u_{1},u_{2},u_{3})(\mathbf{x})$, which determines symmetric 3-by-3 tensor for the linearized strain $\boldsymbol{\varepsilon } =\{\varepsilon _{ij}\}_{i,j=1}^{3}( \mathbf{x})$ by

16 $$ \varepsilon _{ij}(\mathbf{u}) =\frac{1}{2}\Bigl( \frac{\partial u_{i}}{\partial x_{j}} + \frac{\partial u_{j}}{\partial x_{i}} \Bigr), \quad i,j=1,2,3, $$

symmetric 3-by-3 tensor for the deviatoric stress $\mathbf{T}^{*} =\{T_{ij}^{*}\}_{i,j=1}^{3} (\mathbf{x})$ and the first stress invariant $p(\mathbf{x})$, which together satisfy the volumetric-deviatoric decomposition (9), the equilibrium equation

17 $$ -\sum _{j=1}^{3} \frac{\partial }{\partial x_{j}} T_{ij}^{*} - \frac{1}{3} \frac{\partial p}{\partial x_{i}} =f_{i},\quad i=1,2,3, \quad \text{in $\Omega $}, $$

and the one-parametric constitutive response equation (11) regularized according to (12) as

18 $$ \boldsymbol{\varepsilon }(\mathbf{u})^{*} =E_{1} \mathbf{T}^{*},\quad \frac{1}{3} {\mathrm{tr}}\boldsymbol{\varepsilon }(\mathbf{u}) =E_{4} \mathcal{F}[B(p)], $$

where the volumetric-deviatoric decomposition (10) of the strain was used with ${\mathrm{tr}}\boldsymbol{\varepsilon }(\mathbf{u}) =\sum _{i=1}^{3} \varepsilon _{ii}(\mathbf{u}) ={\mathrm{div}}(\mathbf{u})$.

It is worth noting that, if the modulus $E_{4}\to 0$, then the latter equation in (18) implies ${\mathrm{div}}(\mathbf{u}) =0$, and together with (17) the limit relations describe the Stokes system for the incompressible solid:

$$ -\frac{1}{2E_{1}} \Delta \mathbf{u} -\frac{1}{3} \nabla p =\mathbf{f}, \quad {\mathrm{div}}(\mathbf{u}) =0\quad \text{in $\Omega $}. $$

The governing equations (16)–(18) are augmented by the mixed boundary conditions: the Dirichlet condition for the clamp

19 $$ \mathbf{u} =\mathbf{0}\quad \text{on $\Gamma _{\mathrm{D}}$}, $$

and the Neumann type condition for the traction

20 $$ \mathbf{T}^{*}\mathbf{n} +\frac{1}{3} p \mathbf{n} =\mathbf{g} \quad \text{on $\Gamma _{\mathrm{N}}$}, $$

where $\mathbf{T}^{*}\mathbf{n} =\sum _{j=1}^{3} T_{ij}^{*}n_{j}$ implies the matrix-vector multiplication.

Now we provide a variational formulation to the nonlinear boundary value problem (16)–(20). In the following $\mathbb{R}^{3\times 3}_{\mathrm{sym}}$ denotes 3-by-3 symmetric tensors. Let $\mathbf{f}\in L^{2}(\Omega ; \mathbb{R}^{3})$ and $\mathbf{g}\in L^{2}(\Gamma _{\mathrm{N}}; \mathbb{R}^{3})$ be given. Find the triple comprised of functions $\mathbf{u}\in H^{1}(\Omega ; \mathbb{R}^{3})$ with $\mathbf{u} =\mathbf{0}$ on $\Gamma _{\mathrm{D}}$, $\mathbf{T}^{*}\in L^{2}(\Omega ; \mathbb{R}^{3\times 3}_{\mathrm{sym}})$ with ${\mathrm{tr}}\mathbf{T}^{*} =0$ and $p\in L^{2}(\Omega ; \mathbb{R})$, such that they satisfy the following variational equations:

21 $$ \int _{\Omega } \Bigl( \mathbf{T}^{*}\cdot \boldsymbol{\varepsilon } ( \mathbf{v})^{*} +\frac{1}{3} p\, {\mathrm{div}}(\mathbf{v}) \Bigr) \,d \mathbf{x} =\int _{\Omega } \mathbf{f}\cdot \mathbf{v} \,d\mathbf{x} + \int _{\Gamma _{\mathrm{N}}} \mathbf{g}\cdot \mathbf{v} \,dS_{\mathbf{x}}, $$

22 $$ \int _{\Omega } \bigl( E_{1} \mathbf{T}^{*} -\boldsymbol{\varepsilon }( \mathbf{u})^{*} \bigr)\cdot \mathbf{S}^{*} \,d\mathbf{x} =0,\quad \int _{\Omega } \Bigl( E_{4} \mathcal{F}[B(p)] -\frac{1}{3} {\mathrm{tr}} \boldsymbol{\varepsilon }(\mathbf{u}) \Bigr) q \,d\mathbf{x} =0 $$

for all admissible test functions $\mathbf{v}\in H^{1}(\Omega ; \mathbb{R}^{3})$ such that $\mathbf{v} =\mathbf{0}$ at $\Gamma _{\mathrm{D}}$, $\mathbf{S}^{*}\in L^{2}(\Omega ; \mathbb{R}^{3\times 3}_{\mathrm{sym}})$ such that ${\mathrm{tr}}\mathbf{S}^{*} =0$, and $q\in L^{2}(\Omega ; \mathbb{R})$. The linearized strain tensors $\boldsymbol{\varepsilon }(\mathbf{v})$ and its deviatoric part $\boldsymbol{\varepsilon }(\mathbf{v})^{*}$ are defined according to formulas (16) and (10). Here and in what follows, the dot implies the scalar product of tensors $\mathbf{T}\cdot \mathbf{S} =\sum _{i,j=1}^{3} T_{ij} S_{ij}$ and vectors, respectively, $\mathbf{u}\cdot \mathbf{v} =\sum _{i=1}^{3} u_{i} v_{i}$.

The variational equation (21) is obtained in a standard way after multiplication of the equilibrium equation (17) with $v_{i}$, summing it over $i =1,2,3$ and integrating by parts over $\Omega $ with the help of boundary conditions (19) and (20). The variational equations in (22) are derived from the constitutive equations in (18) after taking the scalar product with the test functions $\mathbf{S}^{*}$ and $q$.

Before starting the well-posedness analysis of the nonlinear equations, we record two results that we shall be using. The Korn–Poincaré inequality is given by

23 $$ \|\mathbf{u}\|^{2}_{L^{2}(\Omega ; \mathbb{R}^{3})} \le C_{\mathrm{KP}} \| \boldsymbol{\varepsilon }(\mathbf{u}) \|^{2}_{L^{2}(\Omega ; \mathbb{R}^{3\times 3}_{\mathrm{sym}})} \quad \text{if }\mathbf{u} = \mathbf{0}\;\text{on }\Gamma _{\mathrm{D}}. $$

Together with (23), uniform continuity of the trace operator leads to the estimate:

24 $$ \|\mathbf{u}\|^{2}_{L^{2}(\Gamma _{\mathrm{N}}; \mathbb{R}^{3})} \le C_{ \mathrm{tr}} \|\boldsymbol{\varepsilon }(\mathbf{u}) \|^{2}_{L^{2}(\Omega ; \mathbb{R}^{3\times 3}_{\mathrm{sym}})} \quad \text{if }\mathbf{u} = \mathbf{0}\;\text{on }\Gamma _{\mathrm{D}}. $$

### Theorem 1

(Well-posedness of the regularized problem)

*For every fixed threshold* $0< \underline{b}< \overline{b}< \infty $, *there exists unique triple* $\mathbf{u}\in H^{1}(\Omega ;\mathbb{R}^{3})$ *with* $\mathbf{u} =\mathbf{0}$ *on* $\Gamma _{\mathrm{D}}$, $\mathbf{T}^{*}\in L^{2}(\Omega ; \mathbb{R}^{3\times 3}_{\mathrm{sym}})$ *with* ${\mathrm{tr}}\mathbf{T}^{*} =0$, *and* $p\in L^{2}(\Omega ; \mathbb{R})$, *which solves the nonlinear variational equations* (21) *and* (22).

*The solution satisfies the following a*-*priori estimates*:

25 $$ E_{1}(1-\alpha E_{1}) \|\mathbf{T}^{*}\|^{2}_{L^{2}(\Omega ; \mathbb{R}^{3\times 3}_{\mathrm{sym}})} +E_{4}(\underline{b} -3E_{4} \overline{b}^{2} \alpha ) \|p\|^{2}_{L^{2}(\Omega ; \mathbb{R})} \le \frac{1}{2\alpha } C(f, g), $$

*with a positive weight* $\alpha < \min \bigl ( 1/E_{1}, \underline{b}/(3E_{4} \overline{b}^{2}) \bigr )$, *and*

26 $$\begin{array}{c} {∥\varepsilon {\left(u\right)}^{*}∥}_{L^{2}(\Omega ;R_{\mathrm{sym}}^{3\times 3})}=E_{1}{∥T^{*}∥}_{L^{2}(\Omega ;R_{\mathrm{sym}}^{3\times 3})},\;{∥\mathrm{tr}\varepsilon (u)∥}_{L^{2}(\Omega ;R)}\leq 3E_{4}\bar{b}{∥p∥}_{L^{2}(\Omega ;R)}, \end{array}$$

*where the constant* $C(f, g) >0$ *is related to the given forces through*

27 $$ C(f, g) :=\|f\|^{2}_{L^{2}(\Omega ; \mathbb{R}^{3})} +C_{\mathrm{tr}} \|g \|^{2}_{L^{2}(\Gamma _{\mathrm{N}}; \mathbb{R}^{3})}. $$

### Proof

We justify the properties of the operator of the system (21) and (22).

*Coercivity.* Testing (21) with $\mathbf{v} =\mathbf{u}$ and (22) with $(\mathbf{S}^{*}, q) =(\mathbf{T}^{*}, p)$, using ${\mathrm{tr}}\boldsymbol{\varepsilon }(\mathbf{u}) ={\mathrm{div}}(\mathbf{u})$ and the lower threshold in (13) we obtain the relations

28 $$\begin{array}{c} \int_{\Omega }\left(E_{1}{∥T^{*}∥}^{2}+E_{4}\underline{b}p^{2}\right)\;dx\leq \int_{\Omega }\left(E_{1}{∥T^{*}∥}^{2}+E_{4}F[B(p)]p\right)\;dx\\ =\int_{\Omega }\left(T^{*}\cdot \varepsilon {\left(u\right)}^{*}+\frac{1}{3}p\;\mathrm{div}(u)\right)\;dx=\int_{\Omega }f\cdot u\;dx+\int_{\Gamma_{N}}g\cdot u\;dS_{x}. \end{array}$$

Inserting $(\mathbf{S}^{*}, q) =(\boldsymbol{\varepsilon } (\mathbf{u})^{*}, { \mathrm{tr}}\boldsymbol{\varepsilon }(\mathbf{u}))$ into (22) and using the Korn–Poincaré inequality (23) for the norm $\|\mathbf{u}\|^{2}_{H^{1}(\Omega ; \mathbb{R}^{3})} :=\|\mathbf{u}\|^{2}_{L^{2}( \Omega ; \mathbb{R}^{3})} +\|\boldsymbol{\varepsilon }(\mathbf{u}) \|^{2}_{L^{2}( \Omega ; \mathbb{R}^{3\times 3}_{\mathrm{sym}})}$ we obtain

29 $$\begin{array}{c} \frac{1}{1+C_{\mathrm{KP}}}{∥u∥}_{H^{1}(\Omega ;R^{3})}^{2}\leq {∥\varepsilon (u)∥}_{L^{2}(\Omega ;R_{\mathrm{sym}}^{3\times 3})}^{2}=\int_{\Omega }\left({∥\varepsilon {\left(u\right)}^{*}∥}^{2}+\frac{1}{3}{\mathrm{tr}}^{2}\varepsilon (u)\right)\;dx\\ =\int_{\Omega }\left(E_{1}T^{*}\cdot \varepsilon {\left(u\right)}^{*}+E_{4}F[B(p)]\mathrm{tr}\varepsilon (u)\right)\;dx. \end{array}$$

The lower estimates in (28) and (29) imply coercivity.

*Boundedness.* Applying the Cauchy-Schwarz, the Korn–Poincaré (23) and the trace (24) inequalities, we derive the upper estimates from (21)

30 $$\begin{array}{c} \int_{\Omega }\left(T^{*}\cdot \varepsilon {\left(v\right)}^{*}+\frac{1}{3}p\;\mathrm{div}(v)\right)\;dx=\int_{\Omega }f\cdot v\;dx+\int_{\Gamma_{N}}g\cdot v\;dS_{x}\\ \leq \left({∥f∥}_{L^{2}(\Omega ;R^{3})}+\sqrt{C_{\mathrm{tr}}}{∥g∥}_{L^{2}(\Gamma_{N};R^{3})}\right){∥\varepsilon (v)∥}_{L^{2}(\Omega ;R_{\mathrm{sym}}^{3\times 3})}, \end{array}$$

and from (22), using the upper threshold in (13), we get (26).

Due to (14) the nonlinear form and evidently the bilinear forms in (21) and (22) are strongly monotone (hence, strictly monotone). The hemi-continuity is provided by the continuity property of the nonlinearity $\mathcal{F}[B(p)]$ stated in Lemma 1. Therefore, by the Browder–Minty theorem there exists a solution $(\mathbf{u}, \mathbf{T}^{*}, p)$, which is unique due to the strict monotonicity.

Expressing the same term in the estimate (28) with that in (30) for $\mathbf{v} =\mathbf{u}$, and applying the weighted Young inequality with a weight $\alpha >0$, we infer

31 $$\begin{aligned} \int\limits_{\Omega } \bigl( E_{1} \|\mathbf{T}^{*}\|^{2} +E_{4} \underline{b} p^{2} \bigr) \,d\mathbf{x} &\le \bigl( \|f\|_{L^{2}( \Omega ; \mathbb{R}^{3})} +\sqrt{C_{\mathrm{tr}}} \|g\|_{L^{2}(\Gamma _{ \mathrm{N}}; \mathbb{R}^{3})} \bigr) \|\boldsymbol{\varepsilon }(\mathbf{u}) \|_{L^{2}(\Omega ; \mathbb{R}^{3\times 3}_{\mathrm{sym}})} \\ &\le \frac{1}{2\alpha } C(f, g) +\alpha \|\boldsymbol{\varepsilon }( \mathbf{u}) \|^{2}_{L^{2}(\Omega ; \mathbb{R}^{3\times 3}_{\mathrm{sym}})}, \end{aligned}$$

with the constant $C(f, g)$ defined in (27). From (26) it follows that

$$ \|\boldsymbol{\varepsilon } (\mathbf{u})\|^{2}_{L^{2}(\Omega ; \mathbb{R}^{3\times 3}_{\mathrm{sym}})} \le E_{1}^{2} \|\mathbf{T}^{*}\|^{2}_{L^{2}( \Omega ; \mathbb{R}^{3\times 3}_{\mathrm{sym}})} +3 (E_{4} \overline{b})^{2} \|p\|^{2}_{L^{2}(\Omega ; \mathbb{R})}, $$

and substituting it into (31) leads to the a-priori estimate (25). This finishes the proof. □

As an important consequence of Theorem 1 we conclude the following.

### Corollary 1

(Feasibility for the reference one-parametric response)

*If the variational solution* $(\mathbf{u}, \mathbf{T}^{*}, p)$ *obtained for the regularized problem* (16)*–*(20) *fulfills the prescribed thresholds by means of*

32 $$ 1-\frac{1}{\underline{b}}\le \frac{p}{\tau _{\mathrm{cr}}}\le 1- \frac{1}{\overline{b}}, $$

*such that* $\mathcal{F}[B(p)] =B(p)$ *according to* (12), *then it satisfies also the* $\lambda _{4}$-*dependent constitutive relation* (11).

In the next section we present an analytical example of the nonlinear elasticity problem satisfying (32).

## The Analytical Example

**Fig. 4:**
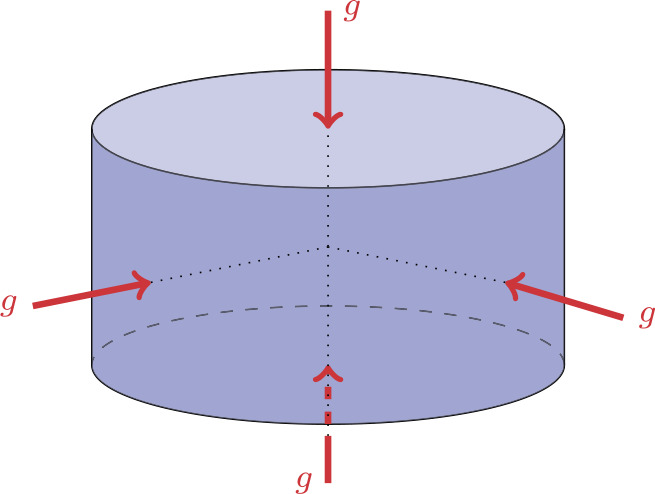
The right circular cylinder $\Omega $ under constant compression $g<0$

We consider an example of constant compression/ extension with given $g\in \mathbb{R}$ applied to a right circular cylinder $\Omega $ (see Fig. 4). In the standard cylindrical coordinates $(r, \theta , z)$, let $\Omega =\{r\le r_{0}, |z|\le z_{0}\}$, where $r_{0}>0$ and $z_{0}>0$ are given. For the body force $\mathbf{f} =0$, the loading is prescribed by

33 $$ \mathbf{T}_{rr} =g\text{ as $r =r_{0}$},\quad \mathbf{T}_{zz} =g \text{ as $z =z_{0}$}. $$

We look for a solution of the form

34 $$ u_{r}(r) =a r,\quad u_{\theta }(r) =b r,\quad u_{z}(z) =a z, $$

with unknown constant $a\in \mathbb{R}$ implying radial and axial stretching and arbitrary $b\in \mathbb{R}$ implying circumferential shear. The corresponding (34) components of the strain tensor are

35 $$\begin{array}{c} \varepsilon_{rr}(u)=u_{r,r}=a,\;\varepsilon_{r\theta }(u)=\frac{1}{2}\left(u_{\theta ,r}-\frac{1}{r}u_{\theta }\right)=0,\;\varepsilon_{\theta \theta }(u)=\frac{1}{r}u_{r}=a,\\ \varepsilon_{zz}(u)=u_{z,z}=a,\;\varepsilon_{rz}(u)=\varepsilon_{\theta z}(u)=0, \end{array}$$

such that the volumetric-deviatoric decomposition of the strain (10) gives the first invariant ${\mathrm{tr}}\boldsymbol{\varepsilon } (\mathbf{u}) =\varepsilon _{rr}(u) + \varepsilon _{\theta \theta }(u) +\varepsilon _{zz}(u) =3a$, and $\boldsymbol{\varepsilon }(\mathbf{u})^{*} =\mathbf{0}$.

According to the first equation in (11) it follows that $\mathbf{T}^{*} =\mathbf{0}$ and the volumetric stress $\mathbf{T} =p/3 \mathbf{I}$ with unknown $p\in \mathbb{R}$. Such a stress tensor satisfies identically the homogeneous equilibrium equations:

$$ -T_{rr,r} -\frac{1}{r} \Bigl( T_{rr} -T_{\theta \theta } \Bigr) =0, \quad -\frac{1}{r} T_{\theta \theta ,\theta } =0,\quad -T_{zz,z} =0. $$

Inserting the expression for $\mathbf{T}$ into the boundary condition (33) we obtain $p/3 =g$. Therefore, the response equations in (11) are solved by

36 $$ a =\frac{1}{3} {\mathrm{tr}}\boldsymbol{\varepsilon } =E_{4} \frac{p}{1 -p/\tau _{\mathrm{cr}}} =E_{4} \frac{3g}{1 -3g/\tau _{\mathrm{cr}}}, $$

for $g\neq\tau _{\mathrm{cr}}/3$, where $\tau _{\mathrm{cr}} :=1/(3E_{4} \lambda _{4})$.

To justify Corollary 1 we conclude that (32) holds for the thresholds prescribed in such a way that

37 $$ 0< \underline{b}\le \frac{1}{1 -3g/\tau _{\mathrm{cr}}},\quad \overline{b} \ge \frac{1}{1 -3g/\tau _{\mathrm{cr}}}. $$

Note that, the left inequality in (37) is attainable only when $1 -3g/\tau _{\mathrm{cr}}>0$ provided $g/\tau _{\mathrm{cr}}>1/3$. Conversely, if (37) holds, then (34) and (36) describe the solution to the regularized problem from Theorem 1.

We note that when $\lambda _{4} =0$ then $\tau _{\mathrm{cr}} =\pm \infty $, and $a =3E_{4} g$ from (36) corresponds to the solution of the linearized problem. In this case, arbitrary thresholds $0<\underline{b}\le 1$ and $\overline{b}\ge 1$ fulfill (37).
